# Neglected Tropical Diseases in Sub-Saharan Africa: Review of Their Prevalence, Distribution, and Disease Burden

**DOI:** 10.1371/journal.pntd.0000412

**Published:** 2009-08-25

**Authors:** Peter J. Hotez, Aruna Kamath

**Affiliations:** 1 Department of Microbiology, Immunology, and Tropical Medicine, The George Washington University, Washington, D.C., United States of America; 2 Sabin Vaccine Institute, Washington, D.C., United States of America; Yale Child Health Research Center, United States of America

## Abstract

The neglected tropical diseases (NTDs) are the most common conditions affecting the poorest 500 million people living in sub-Saharan Africa (SSA), and together produce a burden of disease that may be equivalent to up to one-half of SSA's malaria disease burden and more than double that caused by tuberculosis. Approximately 85% of the NTD disease burden results from helminth infections. Hookworm infection occurs in almost half of SSA's poorest people, including 40–50 million school-aged children and 7 million pregnant women in whom it is a leading cause of anemia. Schistosomiasis is the second most prevalent NTD after hookworm (192 million cases), accounting for 93% of the world's number of cases and possibly associated with increased horizontal transmission of HIV/AIDS. Lymphatic filariasis (46–51 million cases) and onchocerciasis (37 million cases) are also widespread in SSA, each disease representing a significant cause of disability and reduction in the region's agricultural productivity. There is a dearth of information on Africa's non-helminth NTDs. The protozoan infections, human African trypanosomiasis and visceral leishmaniasis, affect almost 100,000 people, primarily in areas of conflict in SSA where they cause high mortality, and where trachoma is the most prevalent bacterial NTD (30 million cases). However, there are little or no data on some very important protozoan infections, e.g., amebiasis and toxoplasmosis; bacterial infections, e.g., typhoid fever and non-typhoidal salmonellosis, the tick-borne bacterial zoonoses, and non-tuberculosis mycobaterial infections; and arboviral infections. Thus, the overall burden of Africa's NTDs may be severely underestimated. A full assessment is an important step for disease control priorities, particularly in Nigeria and the Democratic Republic of Congo, where the greatest number of NTDs may occur.

## Introduction

The neglected tropical diseases (NTDs) are a group of chronic, disabling, and disfiguring conditions that occur most commonly in the setting of extreme poverty, especially among the rural poor and some disadvantaged urban populations [1]. Today, the world's greatest concentration of poverty occurs in sub-Saharan Africa (SSA). According to a recent World Bank analysis, 51% of the population of SSA lives on less than US$1.25 per day, and 73% of the population lives on less than US$2 per day (Table 1) [2]. Previous studies indicate that the NTDs are widespread among the poor in SSA [3]–[6], with the most common NTDs, such as the soil-transmitted helminth (STH) infections, schistosomiasis, lymphatic filariasis (LF), trachoma, and onchocerciasis together affecting more than 500 million people [3], [6]–[8]. Because of their adverse effects on child development, pregnancy outcome, and agricultural worker productivity [1], [3], [6], [7], [9]–[17], the NTDs represent a major reason why the “bottom 500 million” people in SSA cannot escape poverty. Therefore, new and ongoing efforts to control and eliminate the NTDs represent key elements for achieving Africa's Millennium Development Goals (MDGs) for sustainable poverty reduction, including the MDGs to eradicate poverty (MDG 1), promote education (MDG 2), reduce child mortality (MDG 4), improve maternal health (MDG 5), and to combat “other diseases” (MDG 6) [1],[7],[8].

**Table 1:** Poverty in Sub-Saharan Africa.

Percentage of SSA population living on less than US$1.25 per day	51%
Total SSA population living on less than $1.25 per day	390.6 million
Percentage of world's population living on less than US$1.25 per day in SSA	28%
Percentage of SSA population living on less than US$2 per day	73%
Total SSA population living on less than $2 per day	556.7 million
Percentage of world's population living on less than US$2 per day in SSA	22%

From reference [2].

Specific information on the prevalence, distribution, and disease burden resulting from the NTDs in SSA would provide a basis for prioritizing control strategies as a means to address the MDGs. Over the last decade, geographic information systems (GIS) and remote sensing (RS) have facilitated a deeper understanding of the prevalence and distribution of NTDs, particularly for helminth infections in SSA [4], [5], [18]–[21]. Simultaneously, a fresh assessment of the chronic and subtle morbidities caused by NTDs has highlighted a previously underappreciated disease burden [22],[23]. Here, we review current knowledge on the prevalence, distribution, and disease burden resulting from NTDs in SSA, focusing on aspects particular to the region. The review of the literature was conducted using the online database PubMed from 2003 to 2008 with the Medical Subject Headings (MeSH), the specific diseases listed as neglected tropical diseases on the *PLoS Neglected Tropical Disease* Web site (http://www.plosntds.org/static/scope.action), and the geographic regions and countries of SSA. Reference lists of identified articles and reviews were also hand searched as were databases from the World Health Organization (WHO, http://www.who.int), including the WHO's *Weekly Epidemiological Record*.

## Burden and Geographic Distribution of Disease

Table 2 ranks the major NTDs in SSA by their estimated prevalence, the percentage of the population infected, and the percentage of the world's cases found in the region, while Table 3 ranks the countries with the highest prevalence of each of the NTDs. Helminth infections, especially the STH infections, schistosomiasis, and the filarial infections LF and onchocerciasis, are the most common NTDs in SSA, followed by trachoma and other bacterial infections [3], [21]–[38]. Human African trypanosomiasis (HAT) and leishmaniasis are the most common serious protozoan infections, especially in areas of conflict where these diseases emerge in the setting of inadequate housing and forced migrations [39]–[44]. However, as shown in Table 4, there are more than a dozen important NTDs, including the protozoan infections, amebiasis and toxoplasmosis; bacterial infections such as *Salmonella* infections (both typhoid fever and non-typhoidal salmonellosis), the tick-borne zoonoses, and yaws; and viral infections such as Rift Valley fever, for which there is insufficient information available in order to estimate their prevalence in SSA. Based on global disease burden estimates in disability-adjusted life years (DALYs) published previously by the WHO and other investigators [3], [35], [45]–[48], a range of estimates for the NTDs in SSA is provided in Table 5. DALY estimates for the STH infections and schistosomiasis were obtained by adjusting a wide range of available global estimates according to the percentage of the total number of cases that occur in SSA, while for the other NTDs the disease burdens were quoted directly from WHO estimates. From this analysis it was determined that the total burden of NTDs in SSA is possibly as high as one-half the disease burden caused by malaria and twice the disease burden caused by tuberculosis in SSA (Table 6), suggesting that the NTDs represent a formidable public health challenge in the region. Up to 85% of the NTD disease burden specifically results from helminth infections (Table 6). However, even this high disease burden resulting from helminth infections and other NTDs may represent an underestimate because they do not incorporate the full spectrum of chronic sequelae [22],[23], and because such estimates do not include NTDs listed in Table 4.

**Table 2:** Ranking of Neglected Tropical Diseases (NTDs) in SSA by Prevalence and Distribution.

Disease	Estimated Population Infected in SSA	Estimated % of SSA Population Infected	Estimated % Global Disease Burden in SSA	Reference
Hookworm	198 million	29%^a^	34%^b^	[3],[24]
Schistosomiasis	192 million	25%	93%	[21]
Ascariasis	173 million	25%a	21%^2b^	[3],[24]
Trichuriasis	162 million	24%^a^	27%^b^	[3],[24]
Lymphatic filariasis	46–51 million	6%–9%	37%–44%^c^	[25]–[28]
Onchocerciasis	37 million	5%	>99%	[15],[29]
Active trachoma	30 million	3%	48%	[30]
Loiasis	≤13 million	1%–2%	100%	[31],[32]
Yellow fever	180,000	0.02%	90%	[33],[34]
Human African trypanosomiasis	50,000–70,000 (17,000 new cases annually)	<0.01%	100%	[39],[40]
Leprosy	30,055 (registered prevalence); 21,037 new cases in 2007	<0.01%	14%	[35]
Leishmaniasis (visceral)	19,000–24,000 new cases annually in Sudan and Ethiopia	<0.01	ND	[41]–[44]
Dracunculiasis	9,585	<0.01%	100%	[36]
Buruli ulcer	>4,000	<0.01%	57%	[37],[38]

a Based on reported 2003 population of 683,330,334 [24]. For all other estimated population prevalence, we use the 2005 value of 764,328,000 published by the United Nations, http://esa.un.org/unpp/, and querying sub-Saharan Africa and 2005, accessed July 29, 2009.

b Calculated from global burden data from [48].

c The lower value is from [3],[26],[27]; the higher value from [25].

**Table 3:** Geographic Distribution and Estimated Burden of the Major Helminth NTDs in SSA.

Disease (Number of Cases in SSA)	Country with Highest Prevalence	Country with Second Highest Prevalence	Country with Third Highest Prevalence	Country with Fourth Highest Prevalence	Reference
Hookworm infection (198 million)	Nigeria 38 million	DR Congo 31 million	Angola and Ethiopia 11 million cases each	Cote d'Ivoire 10 million	[24]
Schistosomiasis (192 million)	Nigeria 29 million	Tanzania 19 million	DR Congo and Ghana 15 million cases each	Mozambique 13 million	[21]
Ascariasis (173 million)	Nigeria 55 million	Ethiopia 26 million	DR Congo 23 million	South Africa 12 million	[24]
Trichuriasis (162 million)	Nigeria 34 million	DR Congo 26 million	South Africa 22 million	Ethiopia 21 million	[24]
Lymphatic filariasis (382–394 million at risk)	Nigeria 106 million at risk	DR Congo 49 million at risk	Tanzania 31 million at risk	Ethiopia 30 million at risk, Kenya 29 million at risk	[28]
Trachoma (30 million)	Ethiopia 10.3 million	Sudan 3.6 million	Tanzania 2.1 million	Kenya and Niger 2.0 million cases each	[30]
Yellow fever (180,000)	Cote d'Ivoire 16 reported cases in 2006	Mali 5 reported cases in 2006	Cameroon, CAR, Ghana, and Guinea 1 case each in 2006		[33],[34]
Human African trypanosomiasis (50,000–70,000)	DR Congo 10,369	Angola 2,280	Sudan 1,766	Congo 839	[39]
Leprosy (30,055)	DR Congo 6,502	Nigeria 5,381	Ethiopia 4,611	Mozambique 1,830	[35]
Leishmaniasis (visceral) (19,000–24,000 new cases)	Sudan 15,000–20,000 new cases	Ethiopia 4,000 new cases	Kenya and Uganda not determined		[41]–[44]
Dracunculiasis (9,585)	Sudan 5,815	Ghana 3,358	Mali 313	Nigeria and Niger<100 cases each	[36]
Buruli ulcer (>4,000)	Cote d'Ivoire 2,000	Benin and Ghana 1,000 each			[37]

**Table 4:** Major NTDs with No Regional Prevalence or Incidence Estimates in SSA.

Helminth Infections	Protozoan Infections	Bacterial Infections	Viral Infections	Other Conditions
Strongyloidiasis	Amebiasis	Bovine tuberculosis	Dengue fever	Podoconiosis
Taeniaisis	Toxoplasmosis	Tick-borne relapsing fever	Rift Valley fever	
Paragonimiasis		African tick-bite fever	Chikungunya	
Oesophagostomiasis		Typhoid fever	Rabies	
		Non-typhoidal salmonellosis		
		Yaws		

**Table 5:** Disease Burden (DALYs) in SSA Resulting from the NTDs.

Disease	Estimated Global Disease Burden in DALYs	Estimated % Disease Burden in SSA	Estimated SSA Disease Burden in DALYs	Reference
Hookworm	1.5–22.1 million	34%	0.5–7.5 million	[46]–[48]
Schistosomiasis	1.7–4.5 million	93%	1.6–4.2 million	[21],[45],[47]
Ascariasis	1.8–10.5 million	21%	0.4–2.2 million	[46]–[48]
Lymphatic filariasis	5.8 million	35%	2.0 million	[45]
Trichuriasis	1.8–6.4 million	27%	0.5–1.7 million	[46]–[48]
Human African trypanosomiasis	1.5 million	100%	1.5 million	[45]
Trachoma	2.3 million	52%	1.2 million	[45]
Onchocerciasis	0.5 million	99%	0.5 million	[45]
Leishmaniasis	2.1 million	18%	0.4 million	[45]
Leprosy	0.2 million	14%	0.02 million	[35],[45]
Dengue	0.6 million	<1%	0.005 million	[45]
Total NTDs	≤56.6 million	15%–37%	8.6 million–21.2 million	[47]

DALY estimates for STH infections and schistosomiasis were obtained by adjusting a wide range of available global estimates according to the percentage of the total number of cases that occur in SSA, while for the other NTDs the disease burdens were quoted directly from WHO estimates.

**Table 6:** Ranking by Disease Burden (DALYs) and Comparison of Total NTDs with HIV/AIDS, Tuberculosis, and Malaria.

Disease	Disease Burden in SSA (DALYs)	Reference
HIV/AIDS	64.0 million	[45]
Malaria	40.9 million	[45]
NTDs	8.6–21.2 million	
Helminth infections	5.4–18.3 million	
Tuberculosis	9.3 million	[45]

### Helminth Infections

#### STH infections (hookworm infection, ascariasis, trichuriasis)

Since the global prevalence of STH infections was first estimated by Stoll over 60 years ago [31], the overall prevalence of the STH infections is believed to have remained relatively constant in SSA, whereas it has diminished elsewhere in the developing world [24]. Today, between one-quarter and one-third of SSA's population is affected by one or more STH infections [24], with children, especially school-aged children, disproportionately affected. Of the estimated 181 million school-aged children in SSA, almost one-half (89 million) are infected with hookworm, ascariasis, trichuriasis, or some combination of these STH infections [4]. Typically, children exhibit higher STH intensities than any other single population [4] and as a result suffer from profound physical and mental deficits [6], [10], [49]–[51]. Such deficits partially account for their high disease burden in SSA. Moreover, in Kenya (and presumably elsewhere), these effects also translate into increases in school absenteeism and reduced school performance [14].

*Hookworm infection (“hookworm”)*. Hookworm is the most common STH infection and the most common NTD in SSA (Tables 2 and 3). It is also one of the most important in terms of disease burden, accounting for up to one-third of the total burden from NTDs in SSA (Table 5). Based on previous estimates derived in 2002 [24], it is estimated that 198 million people in SSA are infected with hookworm (29% of the region's population), including 40–50 million school-aged children [4],[5]. Approximately one-third of the world's hookworm today occurs in SSA (Table 2) [3], with the greatest number of cases occurring in Nigeria (38 million) and the Democratic Republic of Congo (DRC, 31 million), followed by Angola, Ethiopia, and Cote d'Ivoire (10–11 million) (Table 3). Hookworm is the most widely distributed NTD in SSA and it is pervasive throughout the region (including both rural and urban areas) except in some parts of extreme southern Africa [4],[5]. Two areas are particularly notable for their high hookworm prevalence and intensity compared to other helminth infections, namely coastal regions [52], and areas of extremely high temperatures (where land surface temperatures exceed 37–40°C), including those near the Sahel such as Cameroon [18], Chad [19], and Mali [53],[54]. Through GIS/RS such information can be used to generate predictive maps of areas of high hookworm prevalence as well as their geographic overlap with potential co-infections [4],[5]. For instance, it is estimated that approximately 90% of the 50 million school-aged children with hookworm are at risk for coincident co-infection with falciparum malaria [5],[55]. Both *Necator americanus* and *Ancylostoma duodenale* are found in SSA, with the former representing the predominant hookworm species [56]. Because they cause intestinal blood loss, hookworm is a leading cause of iron deficiency anemia in the region [57]. Among school-aged children in Zanzibar, 35% of iron deficiency anemia and 73% of severe anemia was attributable to hookworm [58], while in Kenya and elsewhere in Africa, hookworm also is an important cause of anemia among preschool children [59],[60]. Hookworm has also been recognized as an important cause of anemia and morbidity in women of reproductive age in SSA, especially among pregnant women [57],[61]. At any given time, almost 7 million pregnant women in SSA (up to one-third of pregnant women in the region) are infected with hookworm [17]. Hookworm's high disease burden in SSA reflects its importance as a cause of maternal and child anemia (Table 5).

*Ascariasis and trichuriasis*. The highest intensity *Ascaris* and *Trichuris* infections occur in school-aged children [4]. It is estimated that 173 million and 162 million people are infected in SSA with *Ascaris* and *Trichuris*, respectively, with 36 million school-aged children infected with ascariasis and 44 million with trichuriasis [4]. For both infections the largest number of cases occurs in Nigeria, where co-infections with hookworm are common [62]. Tens of millions of cases are also found in Ethiopia, DRC, and South Africa (Table 3). Compared to hookworm, both ascariasis and trichuriasis exhibit a more patchy distribution in SSA, with the highest prevalence occurring in equatorial Central and West Africa, eastern Madagascar, and southeast Africa [4]. In contrast to the high rates of ascariasis and trichuriasis in South Africa [63],[64], hookworm is less common except in KwaZulu-Natal [52]. Moreover, higher prevalence rates of ascariasis and trichuriasis are often present in Africa's urban areas compared to rural areas, unlike hookworm, which is more evenly distributed [4]. These observations may reflect the ability of *Ascaris* and *Trichuris* eggs to survive in urban environments, so that increased urbanization in SSA may promote emergence of ascariasis and trichuriasis in the future.

#### Other STH infections

Strongyloidiasis causes diarrhea and malnutrition in SSA, although there is little information on its distribution or disease burden (Table 4), in part because of the difficulties in diagnosing this infection. In one study, strongyloidiasis accounted for 5.3% of diarrhea in malnourished Nigerian children [65]. Two other intestinal nematode infections are focally endemic. *Oesophagostomum bifurcum* is common in northern Ghana and Togo [66], and *Ternidens deminutus* (the “false hookworm”) occurs in Zimbabwe [67].

#### Schistosomiasis and other platyhelminthiases

Of the world's 207 million estimated cases of schistosomiasis, 93% occur in SSA (192 million) (Table 2), with the largest number in Nigeria (29 million) followed by United Republic of Tanzania (19 million), and DRC and Ghana (15 million each) [21] (Table 3). Approximately 76% of the population in SSA lives near rivers, lakes, and other water bodies contaminated with snail intermediate hosts [21], [68]–[70]. Those living near dam reservoirs are at particular risk [21], and SSA has several examples where the infection has emerged or where there has been a dramatic rise in the prevalence of schistosomiasis as a result of irrigation project construction [21],[71]. Climate change and global warming may also be factors [72].

The highest prevalence and intensities of human schistosomiasis occur in school-aged children, adolescents, and young adults who also suffer from the highest morbidity and mortality. There are two major forms of schistosomiasis found in SSA. Approximately two-thirds of the schistosomiasis cases are due to infection caused by *Schistosoma haematobium*, which represents an important cause of severe urinary tract disease [73]. In 2000, van der Werf et al. estimated that 70 million and 32 million individuals out of 682 million people in SSA had experienced hematuria and dysuria, respectively, within the last two weeks [73]. *S. haematobium* produces bladder wall pathology in approximately 18 million people in SSA, and 10 million people suffer from hydronephrosis [73]. Renal failure accounts for a large percentage of the estimated 150,000 deaths from urinary tract schistosomiasis in SSA, and there is also a significant association between major bladder wall pathology and squamous cell carcinoma [74]. A significant percentage of women and men with urinary schistosomiasis acquire genital ulcers and other lesions [22]. In the former, urogenital schistosomiasis is a significant cause of poor reproductive health, including sexual dysfunction and infertility [75]. Genital schistosomiasis also promotes the horizontal transmission of HIV/AIDS in SSA [76]. Intestinal schistosomiasis from *S. mansoni* causes most of the remaining cases in SSA. An estimated 4.4 million people with *S. mansoni* have bloody diarrhea and bowel ulceration, and 8.5 million develop hepatomegaly and/or associated periportal liver fibrosis, portal hypertension, and hematemesis from *S. mansoni* infection, with approximately 130,000 deaths [22],[73]. *S. intercalatum* causes a second form of intestinal schistosomiasis, but with a restricted distribution in West and Central Africa [77].

In addition to the organ-specific pathology described for both *S. haematobium* and *S. mansoni* infections, there is increasing evidence for more generalized morbidity resulting from the chronic inflammation of these long-standing infections [22],[23]. Among the most important are anemia of chronic inflammation and iron deficiency anemia, growth stunting and malnutrition, fatigue and diminished physical fitness, and impaired cognitive development [22],[23]. The current disease burden calculations for schistosomiasis range between 1.7 and 4.5 million DALYs lost annually (1.6 and 4.2 million DALYs in SSA) (Table 5), but these current estimates do not fully consider the general morbidities outlined above. It has been suggested that the true disease burden for schistososomiasis may be several fold higher than previous estimates [22], possibly making this infection the most important NTD in SSA. In addition to *S. haematobium* and HIV co-infections [76], *S. mansoni* and hookworm co-infections are common in SSA and can lead to severe anemia [78]–[80]. A relationship has also been proposed between schistosomiasis and malaria [81].

Among the other platyhelminth infections in SSA, paragonimiasis has been reported from eastern Nigeria and southwestern Cameroon [82], although there are no estimates of the number of cases. Cysticercosis caused by the pork tapeworm *Taenia solium* is a major risk factor for epilepsy in SSA except in Muslim areas [83], and it is hyperendemic in Burundi and elsewhere in eastern Africa [84], southern Africa [85],[86], and Cameroon [87]. It has been suggested that cysticercosis may account for the presence of subcutaneous nodules that erroneously have been linked with onchocerciasis and may be responsible for seizures following mass drug administration with anthelminthics [88]. However, the full disease burden of cysticercosis in SSA remains largely unstudied. Both taeniasis caused by the beef tapeworm *Taenia saginata* and cystic echinococcosis are highly prevalent in East Africa, especially in Ethiopia and Sudan [89],[90].

#### Filarial infections (LF, onchocerciasis, loiasis, and dracunculiasis)

*LF*. Approximately 40% of the world's 120 million cases of LF occur in SSA (approximately 46–51 million cases) (Table 2) [3], [7], [25]–[28],[91],[92], with an estimated 382–394 million people at risk of infection, including 176 million children [91],[93]. In the 39 countries where LF occurs in SSA, the greatest numbers of people at risk of infection live in Nigeria, followed by DRC, Tanzania, Ethiopia, and Kenya (Table 3). All of the LF cases in SSA are caused by *Wuchereria bancrofti*, which are transmitted by a variety of culicine and anopheline mosquitoes [92]. *W. bancrofti* infection produces a wide range of clinical manifestations, including hydrocele and lymphoedema as the most clinically obvious because of the associated disability, disfigurement, and stigma [94]. Based on global estimates that 12.5% of LF infections are estimated to result in lymphedema and 20.8% in hydrocele [93], there are approximately 5 million cases of lymphedema and 8 million cases of hydrocele in SSA. The estimated 2.0 million DALYs lost annually from LF rank it third or fourth behind hookworm and schistosomiasis (and possibly ascariasis) as the most important NTD in SSA (Table 5). LF is also associated with huge economic losses, impairing economic activity up to 88% [9], and causes almost US$1 billion in annual losses, mostly resulting from the disability linked to hydrocele in men [95],[96]. In addition to LF, endemic non-filarial elephantiasis (podoconiosis) is widespread in SSA, with the areas of highest prevalence in the highlands of East Africa as well as in some West African countries [97].

*Onchocerciasis*. More than 99% of the estimated 37 million cases of onchocerciasis occur in SSA (Table 2) [15], distributed in a wide belt that extends from Senegal in the west to Ethiopia in the east and from Mali in the north to Angola and Malawi in the south [15],[29]. Based on rapid epidemiological mapping of onchocerciasis (REMO), a non-invasive and practical tool for distribution and disease prevalence, it is estimated that the mean infection rate among the 19 countries targeted by the African Programme for Onchocerciasis Control (APOC) is 38.2% with 87 million persons at risk for contracting the infection [15]. In SSA, the clinical features of the disease vary from the savanna form, more common in West Africa and associated with high rates of blindness, to the rainforest form more common in Central and East Africa in which high rates of onchocera skin disease (OSD) are characterized by severe pruritus and disfigurement (also known as “troublesome itching”) [29]. In hyperendemic communities where the prevalence of onchocerciasis exceeds 60%, blindness can occur in 10% or more of some savanna populations, while OSD can affect more than 50% of some rainforest communities [29]. Both forms are linked with high disability as well as severe socioeconomic consequences. It is estimated that 40% of the DALYs lost from onchocerciasis result from blindness, while 60% are from OSD [29].

*Loiasis*. The African eyeworm has a high prevalence in rainforest areas of low socioeconomic status and in some savanna regions [32]. The infection is common in Angola, Benin, Cameroon, Central African Republic, Congo, DRC, Equatorial Guinea, Gabon, Nigeria, and Sudan [32]. *Loa* infection is associated with Calabar swellings that result from filarial migrations in the subcutaneous tissues, but the greatest concern about the infection is the risk associated with ivermectin treatments for onchocerciasis co-infections [98]. Angola, Cameroon, and DRC exhibit the highest rates of co-endemicity and consequently, represent the highest risk areas for serious adverse events during mass drug administration [98]. A rapid assessment method based on a clinical history of eyeworm infection and known as RAPLOA is in use to evaluate local prevalence of loiasis, as well as a spatial model based on environmental factors [98].

*Dracunculiasis*. All of the world's cases of dracunculiasis occur in SSA. In 2007, only 9,585 cases were reported (Table 2), a 99% reduction in the number of cases since most endemic countries began to report using village-based surveillance systems [36]. During 2007, Sudan (5,815) and Ghana (3,358) accounted for 96% of the total cases, with the remainder in Mali, Niger, and Nigeria (Table 3) [36]. Mali experienced two unexpected outbreaks during this period [99].

### Protozoan Infections

HAT and leishmaniasis are the major protozoan infections in SSA, accounting for almost 2 million DALYs lost annually (Table 5). Amebiasis and toxoplasmosis are also highly endemic in SSA, but there are few estimates of their prevalence, incidence, or disease burden (Table 4).

#### HAT

Through stepped-up public health control efforts over the last decade, the major endemic countries in SSA have made great strides in reducing the number of cases of HAT from 300,000–500,000 cases to approximately 50,000–70,000 cases, along with 17,000 new cases occurring annually (Table 2) [39]. Approximately 90% of the cases are caused by *Trypanosoma brucei gambiense*, a cause of chronic Gambian HAT [100], with most of the new cases occurring in DRC (10,369 new cases in 2004), followed by Angola, Sudan, Republic of Congo, and Central African Republic (Table 3). The major reductions in Gambian HAT are a result of interruptions in conflict [39]. Despite these gains, outbreaks of Gambian HAT occurred in Angola, DRC, and Sudan in 2005 [40]. HAT caused by *T. b. rhodesiense* accounts for the remaining cases of HAT in SSA, with most of the new cases occurring in Malawi, Uganda, and the United Republic of Tanzania [39]. Rhodesian HAT is a zoonosis transmitted from cattle and other mammals, which produces an acute and fulminating sleeping sickness [39],[100]. Populations in the age group between 15 and 45 years and living in remote rural areas are considered especially vulnerable to both forms of HAT [39]. Currently, the only country with both Gambian and Rhodesian HAT is the nation of Uganda, but to date each focus is geographically separated [101].

#### Leishmaniasis

Both visceral and cutaneous forms occur in SSA, with the former producing serious disease associated with high mortality. Most of the cases of visceral leishmaniasis (VL) occur in the Horn of Africa, i.e., the East African countries of Sudan, Eritrea, Ethiopia, Kenya, and Somalia [43], with most of them caused by *Leishmania donovani* (Table 3), although some cases are caused by *L. infantum* [42]. Because many cases occur in areas of conflict and forced human migrations, the exact number occurring in East Africa is not well established, nor is the disease burden. During the 1980s, an estimated 100,000 people died as a result of VL epidemics [42]. In Sudan alone, as a result of long-standing civil war, hundreds of thousands of cases occurred with death rates exceeding 50% in some areas [43]. Today, the continuing widespread conflict in these countries has destroyed housing and health care infrastructure, and the resultant forced migrations to endemic areas still promote the emergence of VL [42]. Adding to the problem is widespread malnutrition as a result of drought, which increases susceptibility to infection and contributes to the progression of disease [42]. The highest incidence of the disease occurs in Sudan (especially near part of its border with Ethiopia, where 15,000–20,000 new cases occur annually), followed by Ethiopia with approximately 4,000 new cases (Table 2) [41],[43]. The Pokot territory of Kenya and Uganda is also endemic [102]. In these areas VL is considered primarily an anthroponotic infection [103],[104]. VL is also an important opportunistic infection associated with HIV/AIDS in East Africa [41], especially in the Tigray region on the Sudan–Eritrea border where soldiers and seasonal workers sleep outdoors in sandfly-infested areas [43]. Cutaneous leishmaniasis caused by infection with *Leishmania major* is also endemic in parts of SSA including West Africa, where there has been a major increase in the number of cases near Ougadougou, the Burkina Faso capital [41], and in Sudanese refugee camps in Chad [43].

#### Amebiasis

The epidemiology of amebiasis is poorly understood in SSA because few studies differentiate true infection caused by *Entamoeba histolytica* versus infection resulting from the non-pathogenic variant, *E. dispar* [105]. Based on seroprevalence studies in Sudan, Cote d'Ivoire, and South Africa, however, the distribution of amebiasis is believed to be widespread [105]. Amebiasis has also been reported from Nigeria [106]. In South Africa it has been observed that invasive *E. histolytica* infection is associated more commonly with amebic liver abscess than colitis [105].

#### Toxoplasmosis

The study of toxoplasmosis has also been neglected in SSA, although investigations conducted in Sudan [107] and Burkina Faso [108] indicate a high seroprevalence among pregnant women, while studies in West Africa [109] indicate a high seroprevalence in children. Toxoplasmosis is considered a common AIDS defining illness in Ethiopia [110], Nigeria [111], and presumably elsewhere in SSA.

### Bacterial Infections

The major bacterial NTDs are trachoma, mycobacterial infections, tick-borne zoonoses, typhoid fever and non-typhoidal salmonellosis, and yaws. With the exception of trachoma, data on the regional prevalence and/or incidence of these diseases area severely lacking, as are disease burden estimates in DALYs (Table 4).

#### Trachoma

Trachoma is the leading cause of infectious and preventable blindness worldwide [112]–[114], and the most important bacterial infection in SSA. Of the 63 million cases of active trachoma globally (although some estimates indicate 84 million cases worldwide), 48% occur in SSA (30 million) (Table 2) [115]. Nearly half of the global disease burden of active trachoma and a quarter of end-stage trichiasis are concentrated in ten countries alone, with six of these located in SSA [116]. Geographically, trachoma is distributed mainly in the savannah areas of East and Central Africa and the Sahel of West Africa [112]. Ethiopia has the largest number of cases (10.2 million), followed by Sudan (3.6 million) and Tanzania, Kenya, and Niger (2.0–2.1 million each) (Table 3). Approximately one-half of the 2.3 million global trachoma disease burden in DALYs is attributed to SSA (Table 4). Like the other NTDs, trachoma is sustained in a setting of poverty. Additional risk factors for trachoma transmission include crowding and household clustering, insufficient access to water, poor sanitation and facial hygiene, and young children as the reservoir of infection [115]. For instance, in southern Sudan, where more than 90% of the people live on less than US$1 per day, and only 27% have access to water and 16% to improved sanitation conditions, the prevalence of trachoma is ranked among the highest worldwide [117],[118]. Hyper-endemic areas in SSA have been found to have an earlier age of onset of trichiasis [118], while women are two to four times more likely to have trichiasis due to increased exposure to young children [116],[119]. Dry zones with limited water accessibility and hot lowlands (altitude <3,000 m) with dense fly populations also promote transmission [112],[120]. Post-conflict conditions, namely in Sudan and Rwanda, have been assessed, but with differing conclusions on their impact on the prevalence of blindness. In southern Sudan, the prevalence of blindness (4.1%) is four times greater than that of the rest of Africa [121].

#### Mycobacterial infections

*Buruli ulcer*. Of the estimated 7,000 cases of Buruli ulcer reported annually [38], more than 4,000 cases occur in SSA (Table 2), with the largest number reported from the West African countries of Cote d'Ivoire (approximately 2,000 cases) and Benin and Ghana (reporting approximately 1,000 cases each) (Table 3) [37]. The greatest risk factors for acquiring Buruli ulcer include residing in an endemic area, close proximity to specific bodies of water, and age less than 15 years [37],[38].

*Leprosy*. In Africa, the number of new cases of leprosy has declined every year since 2001 [35]. At the beginning of 2008, approximately 30,055 cases of leprosy were registered in Africa with 31,037 new cases in 2007 (Table 2) [35]. These represent approximately 14% and 12% of the global prevalence and new cases, respectively (Table 2). In SSA, the highest registered prevalence occurs in DRC (6,502 cases), followed by Nigeria (5,381), Ethiopia (4,611), and Mozambique (1,830) (Table 3) [35]. Similarly, in 2007, the largest number of new cases occured in DRC (8,820), Nigeria (46,650), Ethiopia (4,187), and Mozambique (2,610) [35].

*Bovine tuberculosis*. Tuberculosis is a major opportunistic infection among HIV-infected populations in SSA and it is estimated that 70% (6 million) of the people co-infected with tuberculosis and HIV live in SSA [122]. The proportion of African tuberculosis due to *Mycobacterium bovis* is unknown [123], although it is estimated that approximately 50% of African cattle live in countries without control measures for the disease [122]. In SSA, bovine tuberculosis frequently passes from livestock to wildlife, while human populations become infected through the consumption of raw milk and raw meat (including bushmeat) [122]. The prevalence and incidence of bovine tuberculosis in SSA has not been established.

#### Tick-borne zoonoses

*Tick-borne relapsing fever (TBRF)*. TBRF is an acute febrile illness caused by the spirochaete *Borrelia crocidurae* and transmitted by an *Ornithodoros* tick [124]. If left untreated, patients develop severe relapsing remitting fever, and in some parts of Senegal and elsewhere in West Africa this infection is second only to malaria as the most common cause of outpatient visits [124]. However, detailed information on the precise incidence and distribution of TBRF in SSA is lacking because the disease is underdiagnosed and medical personnel have inadequate awareness of this infection and frequently confuse it with malaria [124].

*African tick-bite fever*. African tick-bite fever is a spotted fever group rickettsiosis caused by *Rickettsia africae* and transmitted by *Amblyomma* ticks [125]. It is known to be endemic in Zimbabwe and South Africa, although based on serologic surveys the infection also occurs elsewhere in SSA [125],[126]. Infection with *R. africae* is common in travelers returning to Europe and North America [125],[126].

#### *Salmonella* and other enteric infections

Although well-publicized outbreaks of typhoid fever (*Salmonella typhi* infection) have been reported from Kenya [127], the overall incidence of culture-proven typhoid fever may be lower than 1/100,000 person years [128]. In SSA, *S. typhi* infection has been noted to occur in association with schistosome infections [129]. Of concern is the emergence of drug-resistant typhoid fever [129],[130]. Similarly, nontyphoidal salmonella (NTS) infection and bacteremia (some of which are caused by drug-resistant strains) is emerging as an important opportunistic infection in individuals infected with HIV and in children [131],[132]. In Malawi and elsewhere in SSA, NTS bacteremia typically presents in children less than 3 years of age, especially those with coincident anemia, malaria, malnutrition, and HIV infection [131]–[133]. Among adults, NTS is most commonly associated with advanced HIV disease [131]. In Malawi, approximately 75% of the cases of NTS are associated with *Salmonella enterica* serovar Typhimrium and 21% with *S. enterica serovar* Enteritidis [131],[132]. *Campylobacter jejuni* has emerged as a common cause of enteritis in Nigeria [134].

#### Yaws

Yaws is a skin disease caused by *Treponema pallidum*, subspecies *pertennae*, and Africa has the highest concentration of the disease [135]. Africa's indigenous pygmies are disproportionately affected, although there are no published estimates of the disease.

### Viral Infections

Arboviral infections, including yellow fever, dengue, Chikungunya and Rift Valley fever [136], and rabies stand out as the most important viral NTDs. As with the bacterial infections, there are few estimates of their incidence or disease burden.

#### Arobviral infections

Approximately 90% of the world's 200,000 cases of yellow fever (and 30,000 deaths) occur in SSA [33]. Especially in Francophone West Africa, transmission of yellow fever is maintained by high densities of *Aedes aegypti* mosquitoes in close proximity to unvaccinated human populations [33]. Yellow fever is underreported, although a laboratory network in the endemic countries of West and Central Africa was established in 2002 in order to strengthen capacity for diagnosis [34]. In 2006, cases of yellow fever were reported in Cameroon, Central African Republic, Cote d'Ivoire, Ghana Guinea, Mali, and Togo [34]. Since 2000, four urban outbreaks have occurred in West Africa, including a 2001 outbreak in Abidjan, Cote d'Ivoire, that required the immunization of 2.6 million people in 12 days [137]. All four dengue viral types have been reported from Africa, although to date no epidemic of dengue hemorrhagic fever has been reported [138]. Epidemics of Chikungunya fever have also been reported in Angola, Burundi, Cameroon, CAR, Kenya, Namibia, Nigeria, Senegal, South Africa, Tanzania, Uganda, and Zimbabwe [125]. Rift Valley fever is a bunyavirus infection transmitted by mosquitoes, which is a problem not only for humans but also for sheep, goats, and cattle. Epidemics and epizootics occur after periods of heavy rains [139]. Epizootics are closely linked to excess rainfall resulting from El Niño and sea surface temperature anomalies in the Indian and Pacific Oceans [139].

#### Rabies

Following India, the greatest number of cases of human rabies transmitted from dogs occurs in SSA [140]. In Limpopo Province (South Africa) alone, 100 cases occurred in 2006 [140]. There are two distinct biotypes in southern Africa, a canid virus and a mongoose virus [141].

## Concluding Statement: Disease Assessment and Control Priorities

Overall, it should be noted that the estimates of infection prevalence and morbidity provided here are, in many cases, based on limited data. Moreover, there is often spatial heterogeneity of the NTDs within large geographic units, i.e., at the regional or country level. This is particularly true for most of the vector-borne NTDs. From the analysis reported here, two nations stand out has having the largest number of NTD cases and possibly disease burden. Nigeria is estimated to have the highest prevalence of helminth infections such as hookworm, schistosomiasis, ascariasis, trichuriasis, and LF in SSA, as well as the second highest registered prevalence of leprosy (Table 3). In addition, arboviral and other zoonotic infections are common in Nigeria, including yellow fever, rabies, and toxoplasmosis [134], while Buruli ulcer is found in the southern and southeastern areas of the country [142]. Similarly, DRC exhibits the highest prevalence of HAT and leprosy in SSA, as well as the second highest prevalence of hookworm infection and trichuriasis (and possibly LF), and the third highest prevalence of schistosomiasis and ascariasis (Table 3). Together, Nigeria and DRC account for approximately one-third of the helminth infections and leprosy in SSA, as well as up to one-fifth of the cases of HAT. A targeted approach for these two countries could make a substantial impact on Africa's overall NTD disease burden.

The information presented here indicates that helminth infections, especially the three major STH infections, schistosomiasis, LF, and onchocerciasis, account for up to 85% of the disease burden caused by NTDs in SSA (Table 6). Given that NTDs may be responsible for as much as one-half and one-third of the region's malaria and HIV/AIDS disease burden, respectively, helminth control should continue to be prioritized by public health experts, health ministries, and global policy makers. In addition, based on revised estimates for schistosomiasis that fully consider the morbidities of chronic infection including effects on anemia, malnutrition, growth stunting, and diminished child development [22],[23], and the observation that many of these same sequelae occur for STH and other helminth infections [4], [5], [50], [51], [57]–[61], the possibility remains that maternal and child helminthiases may increase even further in their disease control priority rankings. There is an urgent need for better direct or indirect methods of estimating the disease burden for the NTDs and other conditions. Disease burden estimates for the major helminthiases and other NTDs (based on DALYs as the major metric) are currently being re-evaluated in an initiative led by the Institute for Health Metrics and Evaluation at the University of Washington and supported by the Bill & Melinda Gates Foundation [143]–[145]. Another high priority is to begin obtaining prevalence, incidence, and disease burden estimates for many of the non-helminth NTDs. Amebiasis and toxoplasmosis stand out as both common and serious protozoan infections for which we have very little information. Similarly, the data available for human *Salmonella* and tick-borne zoonotic bacterial infections are minimal even though some reports suggest they be enormously important. Stepped-up surveillance measures for some of Africa's arboviral infections may provide timely and useful data in the coming years. A full analysis of all Africa's NTDs is an essential step towards prioritizing these conditions relative to ongoing HIV/AIDS and malaria control efforts supported by private foundations and the Group of Eight nations.
